# Plasma biomarkers in patients with age-related sarcopenia: a proteomic exploration and experimental validation

**DOI:** 10.1007/s40520-024-02903-7

**Published:** 2024-12-27

**Authors:** Qinqing Lin, Kangyong Li, Liwei Li, Lichang Guan, Yingtong Zeng, Dake Cai, Jing Zhou, Lishu Xu

**Affiliations:** 1 Department of Geriatric Gastroenterology, Guangdong Provincial People’s Hospital (Guangdong Academy of Medical Sciences), Southern Medical University, Guangzhou, China; 2 Shantou University Medical College, Shantou, Guangdong China; 3 Department of Pharmacy, Guangdong Provincial People’s Hospital (Guangdong Academy of Medical Sciences), Southern Medical University, Guangzhou, China; 4 Guangzhou University of Chinese Medicine, Guangzhou, China; 5 Guangdong Provincial Institute of Geriatrics, Guangdong Provincial People’s Hospital (Guangdong Academy of Medical Sciences), Southern Medical University, Guangzhou, China

**Keywords:** Sarcopenia, Biomarker, PON3, Plasma proteomics, ELISA, Older adult

## Abstract

**Background:**

Various biomarkers associated with sarcopenia have been identified. However, there is a scarcity of studies exploring and validating biomarkers in individuals with age-related sarcopenia.

**Aims:**

This study aimed to investigate the proteome and identify potential biomarkers for age-related sarcopenia.

**Methods:**

Proteomic analysis and experimental validation were conducted using plasma from hospitalized older adults. Sarcopenia diagnosis was based on the Asian Working Group for Sarcopenia 2019 criteria. Data-independent acquisition-based proteomics was performed on plasma from 60 participants, with 30 diagnosed with sarcopenia and 30 without sarcopenia. Differentially expressed proteins (DEPs) were selected and evaluated by Receiver Operating Characteristic (ROC) analysis. Biomarker candidates were further quantitatively validated by enzyme-linked immunosorbent assay (ELISA) utilizing plasma from 6 participants with sarcopenia and 6 without sarcopenia.

**Results:**

A total of 39 DEPs were identified and 12 DEPs were selected for ROC analysis. 8 DEPs were included for ELISA validation based on their predictive performance. Paraoxonase-3 (PON3) consistently showed down-regulation in the sarcopenic group across both methodologies. Insulin-like growth factor-binding protein-2 (IGFBP2) showed inconsistency in the sarcopenic group, with up-regulation observed in proteomic analysis but down-regulation in ELISA.

**Discussion:**

Decline in PON3 may result in an overload of oxidative stress in skeletal muscles and contribute to sarcopenia. Protein modifications of IGFBP2 might exhibit during sarcopenia pathogenesis.

**Conclusions:**

Plasma proteins are implicated in sarcopenia pathogenesis. PON3 is highlighted as a potential biomarker for patients with age-related sarcopenia. Further studies are imperative to gain an in-depth understanding of PON3 and IGFBP2.

**Supplementary Information:**

The online version contains supplementary material available at 10.1007/s40520-024-02903-7.

## Introduction

Sarcopenia is defined as an age-related pathological loss of skeletal muscle, combined with a decline in muscle strength or physical function [1]. It is prevalent in individuals over 60 years old and is associated with increased adverse health events, poor quality of life, and high economic burden [2, 3]. Common case finding methods include calf circumference or SARC-CalF questionnaires [1]. In Asia, the Asian Working Group for Sarcopenia (AWGS) 2019 criteria is widely adopted for sarcopenia diagnosis, with evaluation of muscle strength, physical performance, and muscle mass in clinics [1]. Physical exercise and nutritional support are employed as conventional therapies [4]. In the realm of pharmacotherapy, although numerous interventions are proposed to alleviate muscle aging, none have been successfully translated into definitive treatments for sarcopenia [5]. This underscores the escalating demand for novel intervention targets, especially with the rapid rise in aging population.

As a geriatric syndrome, sarcopenia is associated with multifactorial pathogenesis, including chronic inflammation, oxidative stress, mitochondrial dysfunction, lipotoxicity, and imbalanced protein metabolism [6–8]. However, the molecular mechanisms remain largely unclear. Biomarkers are critical tools for accessing the molecular field, which facilitate disease prediction [9] and diagnosis [10], elucidate underlying mechanisms [11], and pinpoint therapeutic targets [12]. Various biomarkers associated with sarcopenia have been identified, such as the serum creatinine to cystatin C ratio [10], gluconic acid [13], fibroblast growth factor 21 [14], and inflammatory cytokines [15]. Proteomics, a comprehensive profile of expressed proteins, is considered as a robust technique to explore a wider range of potential biomarkers for the complex process of muscle aging [16]. For example, Dlamini et al. found that CD163 was associated with reduced appendicular skeletal muscle mass (ASM) in males but increased ASM in females within a proteomic analysis of a middle-aged South African cohort [17]. Jiang et al. reported druggable proteins, including tumor necrosis factor ligand superfamily member 12 and hepatocyte growth factor, associated with sarcopenia using a Mendelian randomization analysis of proteomic data [18]. Aparicio et al. proposed the diagnostic biomarkers for sarcopenia based on the proteomic profiling of plasma extracellular vesicles [19]. Huemer et al. indicated novel biomarkers associated with the co-occurrence of low muscle mass and high fat mass through plasma proteomics [20]. Furthermore, Ubaida-Mohien et al. revealed altered muscle proteomes associated with disrupted energetic metabolism and pro-inflammatory status in aging muscles [21]. In the proteomic field, data-independent acquisition (DIA) mass spectrometry (MS) is emerging as a widely utilized high-throughput proteomic technology [22] in screening candidate biomarkers of various diseases [23–25]. Since MS identifies proteins by detecting mass spectrum intensities of peptides and is not inherently quantitative [26], quantification strategies are therefore usually carried out for validation of protein biomarkers, among which enzyme-linked immunosorbent assay (ELISA) is one of the most widely utilized methods [27–29]. It should be noted that not all reported sarcopenic biomarkers have been validated. A recent DIA-based proteomic analysis by Wu et al. proposed potential diagnostic proteins for sarcopenia [30], yet these were not further validated. To our knowledge, there is a scarcity of studies that integrate proteomics exploration with biomarker validation for sarcopenia.

To address this gap, we aim to identify and validate potential plasma biomarkers for age-related sarcopenia by integrating DIA-based proteomic analysis with ELISA, which lays the groundwork for further research into sarcopenia pathogenesis and the discovery of potential therapeutic targets.

## Method

### Study design

We conducted a cross-sectional study following the Strengthening the Reporting of Observational Studies in Epidemiology (STROBE) guidelines [31]. Referring to our previous study [32], this study was approved by the ethics committee of Guangdong Provincial People’s Hospital, China (approval no. GDREC20198345H(RI)). All participants provided written informed consent before registration. All procedures followed the institutional guidelines.

To identify potential plasma biomarkers, proteomic exploration and experimental validation were carried out. First, DEPs between participants with and without sarcopenia were identified using DIA-based proteomic analysis. Next, the promising biomarkers were selected based on Receiver Operating Characteristic (ROC) analysis. Subsequently, the selected biomarkers were validated in an independent cohort. The study design flowchart was shown in Supplementary Fig. 1.

### Study population

Our study included inpatients in the Department of Geriatric Gastroenterology of Guangdong Provincial People’s Hospital from November 2021 to June 2022. All subjects were unrelated to each other. Detailed demographic information, medical history, and prescribed medications were recorded by specialists. The inclusion criteria for the sarcopenic group were as follows: 1) age > 60 years; 2) being diagnosed with sarcopenia according to AWGS 2019 criteria [1]. Individuals older than 60 years old without sarcopenia were enrolled in the non-sarcopenic group. The exclusion criteria for both groups were as follows: (1) being physically inactive caused by other diseases (such as stroke, Parkinson’s disease, multiple fractures, and etc.); and (2) being unable to cooperate due to various diseases (such as Alzheimer’s disease).

Multimorbidity was evaluated utilizing age-adjusted Charlson comorbidity index (aCCI), which assigns scores for age and 17 comorbid conditions [33]. Polypharmacy was assessed by recording the number of daily medications taken by each participant for a period of at least three months. Additionally, the duration of hospitalization was recorded.

### Plasma collection and biochemical measurements

After fasting for at least 8 h, venous blood was drawn from the participant in the morning and collected in several EDTA tubes. Tubes were inverted 10 times manually to ensure thorough mixing of the blood with the anticoagulant. Plasma was obtained by centrifugation at 2000 rpm for 10 min at 4 ℃, then transferred into sterile Eppendorf tubes and stored at – 80 ℃ until protein extraction. Additional blood samples were subsequently sent to the Biochemistry Laboratory at Guangdong Provincial People’s Hospital for analysis. Blood lipid profile, comprising triglyceride, total cholesterol, high-density lipoprotein cholesterol (HDL-C), and low-density lipoprotein cholesterol (LDL-C), along with blood inflammatory markers, including interleukin-6 and C-reactive protein, were assessed according to the standard procedures of the laboratory.

### Sarcopenia assessment

Sarcopenia was diagnosed according to the AWGS 2019 criteria [1]. Appendicular skeletal muscle mass (ASM) was measured by bioelectrical impedance analysis (TANITA MC-980MA; Tanitao Corp., Tokyo, Japan). Body composition assessments were uniformly conducted in the morning on fasting participants with empty bladders in a standing position, who were asked to maintain a standing position for 15 min prior to measurement, and were not wearing coats, shoes, or sweaters, in accordance with the instructions of the manufacturer. Appendicular skeletal mass index (ASMI) was derived by dividing ASM by the square of the individual’s height. Muscle strength was assessed by grip strength utilizing the electronic grip dynamometer (CAMRY EH101; Zhongshan Camry Electronic Co., Ltd., Zhongshan, China). The maximum reading of the 2 measurements of the dominant hand was considered as the final grip strength. Physical performance was evaluated by the six-meter walk test. The highest walk speed of 2 tests was recorded. Grip strength and walk test were carried out in the inpatient ward in the morning, in compliance with standard operational protocols. Sarcopenia diagnosis was confirmed with low ASMI (Male: < 7.0 kg/m^2^, Female: < 5.7 kg/m^2^) and low grip strength (Male: < 28 kg, Female: < 18 kg), or with low ASMI and low physical performance (six-meter walk speed: < 1.0 m/s). Participants presenting low ASMI, low grip strength and low physical performance were diagnosed with severe sarcopenia.

### DIA proteomic analysis

A total of 60 plasma samples, consisting of 30 from participants with sarcopenia and 30 from participants without sarcopenia, were used for DIA proteomic analysis. Construction of data-dependent acquisition (DDA) spectrum library and identification of DIA mode using UHPLC-MS/MS were conducted at LC-bio Co., Ltd. In Hangzhou, China. The process commenced with extracting proteins from plasma samples, proceeding with a series of quality evaluations and trypsin digestion to prepare the samples for analysis. Fraction separation was conducted using a gradient elution method on a C18 column. EASY-nLCTM 1200 UHPLC system (Thermo Fisher, Germany) coupled with a Q Exactive HF-X mass spectrometer (Thermo Fisher, Germany) was utilized for proteomics analyses operating in both DDA and DIA modes. Spectra acquired from DDA mode were searched against the homo_sapiens_uniprot_2019.01.18.fasta (containing 169,389 sequences) by Spectronaut-Pulsar (Biognosys) for DDA spectrum library construction. Peptide Spectrum Matches (PSMs) with confidence exceeding 99% were identified. PSMs and proteins with a False Discovery Rate (FDR) of less than 1.0% were retained for further analysis. Raw data acquired from DIA mode were subsequently imported into Spectronaut (Biognosys) software for qualitative and quantitative peptide analysis based on DDA spectrum library. The t-test was applied to analyze the protein quantification results. Proteins with significant quantitative differences, defined as either a fold change > 1.5 (*p* < 0.05) or a fold change < 0.667 (*p* < 0.05), were defined as differentially expressed proteins (DEPs).

### Functional analysis

InterProScan program was used for Gene Ontology (GO) and InterPro (IPR) functional annotation, including Pfam, PRINTS, ProDom, SMART, ProSite, and PANTHER [34]. Protein families and pathways were examined through Clusters of Orthologous Groups (COG) and Kyoto Encyclopedia of Genes and Genomes (KEGG) analysis. DEPs analysis included volcano map, cluster heatmap, and enrichment analysis of GO, KEGG, and IPR [35]. Predictive potential protein–protein interactions were performed using the STRING website (https://cn.string-db.org/).

### Enzyme‑linked immunosorbent assay

Validation of proteins in 12 plasma samples, with 6 from participants with sarcopenia and 6 from those without sarcopenia, was performed utilizing the double-antibody sandwich enzyme-linked immunosorbent assay (ELISA), based on the manufacturer’s suggested protocols. The following ELISA kits were used: IGFBP2, PON3, LRG1, PRDX6, PTGDS, CD163, PRG4, and TRAJ17 (Camilo, Nanjing, China). Preliminary experiments were conducted to ensure the optimal diluted factor of plasma samples. The 96-well plates were read at 450 nm and 630 nm using a Tecan SPARK microplate reader. All protein assays exhibited intraplate variabilities of less than 10%. CurveExpert version 1.4 was used to draw the standard curve and calculate the protein concentrations. GraphPad Prism 9 was used for plotting.

### Statistical analysis

All quantitative data were subjected to the Shapiro–Wilk normality test. Data showing normal distribution were presented as “mean ± standard deviation” and were analyzed using the two-sided Student’s t test. Data exhibiting skewed distribution were presented as "median (interquartile range)" and were analyzed using the Wilcoxon rank-sum test. Categorical variables were analyzed using the Chi-square test or Fisher’s exact test. Spearman’s correlation analysis was performed for the correlations between DEPs and clinical parameters. Statistical significance was defined as* p* < 0.05. Statistical analysis was conducted using R version 4.2.2 and SPSS version 26, unless otherwise specified.

## Result

### Baseline characteristics of participants

A total of 72 participants were enrolled in this study, with 30 with sarcopenia and 30 without sarcopenia participating in the DIA-based proteomic analysis. Experimental validation was then conducted on the remaining 12 participants. The baseline characteristics of 60 participants and 12 participants were presented respectively (Table 1).

**Table 1:** Baseline characteristics of participants with and without sarcopenia for DIA analysis and ELISA validation [32]

Items	DIA analysis cohort			ELISA validation cohort		
	Sarcopenia (n = 30)	Non-sarcopenia (n = 30)	*p*-value	Sarcopenia (n = 6)	Non-sarcopenia (n = 6)	*p*-value
Age, years	91.0 (83.5, 92.0)	71.0 (65.2, 76.2)	< 0.001^a^	78.7 ± 10.8	70 ± 5.4	0.109^b^
Male, n (%)	18 (60)	21 (70)	0.417^c^	4 (67)	4 (67)	1^d^
BMI, kg/m^2^	21.3 ± 3.5	24.5 ± 2.4	< 0.001^b^	21.2 ± 1.4	25.2 ± 3.1	0.017^b^
ASMI, kg/m^2^	5.5 ± 0.9	7.3 ± 0.9	< 0.001^b^	5.8 ± 0.9	7.9 ± 1.3	0.01^b^
Handgrip strength, kg	15.2 (0.0, 21.4)	28.2 (25.2, 35.1)	< 0.001^a^	18.7 ± 10.5	37.4 ± 5.5	0.003^b^
Six-meter Walk speed, m/s	0.4 (0.0, 0.7)	1.2 (1.0, 1.3)	< 0.001^a^	0.9 (0.3, 1.1)	1.2 (1.0, 1.4)	0.037^a^
Triglyceride, mmol/L	1.1 (0.8, 1.3)	1.0 (0.8, 1.7)	0.631^a^	0.9 (0.8, 1.6)	1.2 (1.0, 2.0)	0.2^a^
Total cholesterol, mmol/L	3.8 (3.0, 4.6)	4.6 (3.5, 5.7)	0.019^a^	4.0 (3.3, 4.3)	4.7 (4.4, 5.9)	0.01^a^
HDL-C, mmol/L	1.1 ± 0.3	1.2 ± 0.2	0.032^b^	1.3 ± 0.4	1.1 ± 0.2	0.392^b^
LDL-C, mmol/L	2.1 (1.7, 2.9)	2.9 (2.0, 3.9)	0.044^a^	2.1 ± 0.5	3.2 ± 0.7	0.011^b^
CRP, mg/L	1.6 (0.7, 6.2)	0.8 (0.5, 1.5)	0.022^a^	1.6 ± 1.5	1.2 ± 0.7	0.625^b^
IL-6, pg/mL	5.5 (3.5, 10.5)	2.1 (1.5, 3.7)	< 0.001^a^	3.4 (1.5, 6.0)	3.2 (1.9, 3.7)	0.628^a^
aCCI	8.0 (6.2, 9.0)	6.0 (5.0, 8.0)	0.004^a^	6.0 (5.2, 8.2)	6.5 (3.7, 7.0)	0.746^a^
Number of drugs	9.5 (5.0, 20.7)	4.5 (2.0, 7.0)	< 0.001^a^	10.0 (6.5, 13.5)	6.0 (5.2, 6.7)	0.258^a^
Duration of hospitalization, days	10.0 (6.0, 15.0)	7.5 (6.2, 14.0)	0.582^a^	9.0 (6.5, 11.5)	3.0 (2.2, 3.7)	0.006^a^

The *p-*value was obtained for comparison of sarcopenic and non-sarcopenic groups by following tests: ^a^Rank sum test, ^b^Student’s t-test, ^c^Chi-square test, ^d^Fisher’s exact test

*BMI* body mass index, *ASMI* appendicular skeletal muscle index, *HDL-C* high density lipoprotein cholesterol, *LDL-C* low density lipoprotein cholesterol, *CRP* C-reactive protein, *IL-6* interleukin-6, *aCCI* age-adjusted Charlson comorbidity index

The baseline characteristics of the participants in the DIA analysis cohort were consistent with our previous research [32]. Participants with sarcopenia were significantly older than those without sarcopenia [sarcopenia, 91.0 (83.5, 92.0) vs. non-sarcopenia, 71.0 (65.2, 76.2) years; *p* < 0.001]. Compared to those without sarcopenia, participants with sarcopenia also had significantly lower BMI (sarcopenia, 21.3 ± 3.5 vs. non-sarcopenia, 24.5 ± 2.4 kg/m^2^; *p* < 0.001), total cholesterol level [sarcopenia, 3.8 (3.0, 4.6) vs. non-sarcopenia, 4.6 (3.5, 5.7) mmol/L; *p* = 0.019], HDL-C (sarcopenia, 1.1 ± 0.3 vs. non-sarcopenia, 1.2 ± 0.2 mmol/L; *p* = 0.032) and LDL-C level [sarcopenia, 2.1 (1.7, 2.9) vs. non-sarcopenia, 2.9 (2.0, 3.9) mmol/L; *p* = 0.044], with significantly increased CRP level [sarcopenia, 1.6 (0.7, 6.2) vs. non-sarcopenia, 0.8 (0.5, 1.5) mg/L; *p* = 0.022] and IL-6 level [sarcopenia, 5.5 (3.5, 10.5) vs. non-sarcopenia, 2.1 (1.5, 3.7) pg/mL; *p* < 0.001]. Also, individuals with sarcopenia had higher aCCI [sarcopenia, 8.0 (6.2, 9.0) vs. non-sarcopenia, 6.0 (5.0, 8.0); *p* = 0.004] and number of drugs [sarcopenia, 9.5 (5.0, 20.7) vs. non-sarcopenia, 4.5 (2.0, 7.0); *p* < 0.001] compared with those without sarcopenia.

In the ELISA validation cohort, the age, IL-6 level, CRP level, aCCI, and number of drugs showed no significant differences between the sarcopenic group and non-sarcopenic group. Compared with individuals in the non-sarcopenic group, individuals in the sarcopenic group had lower BMI (sarcopenia, 21.2 ± 1.4 vs. non-sarcopenia, 25.2 ± 3.1 kg/m^2^; *p* = 0.017), total cholesterol level [sarcopenia, 4.0 (3.3, 4.3) vs. non-sarcopenia, 4.7 (4.4, 5.9) mmol/L; *p* = 0.01] and LDL-C level (sarcopenia, 2.1 ± 0.5 vs. non-sarcopenia, 3.2 ± 0.7 mmol/L; *p* = 0.011).

### Overview of DIA-based proteomic analysis

**Fig. 1:**
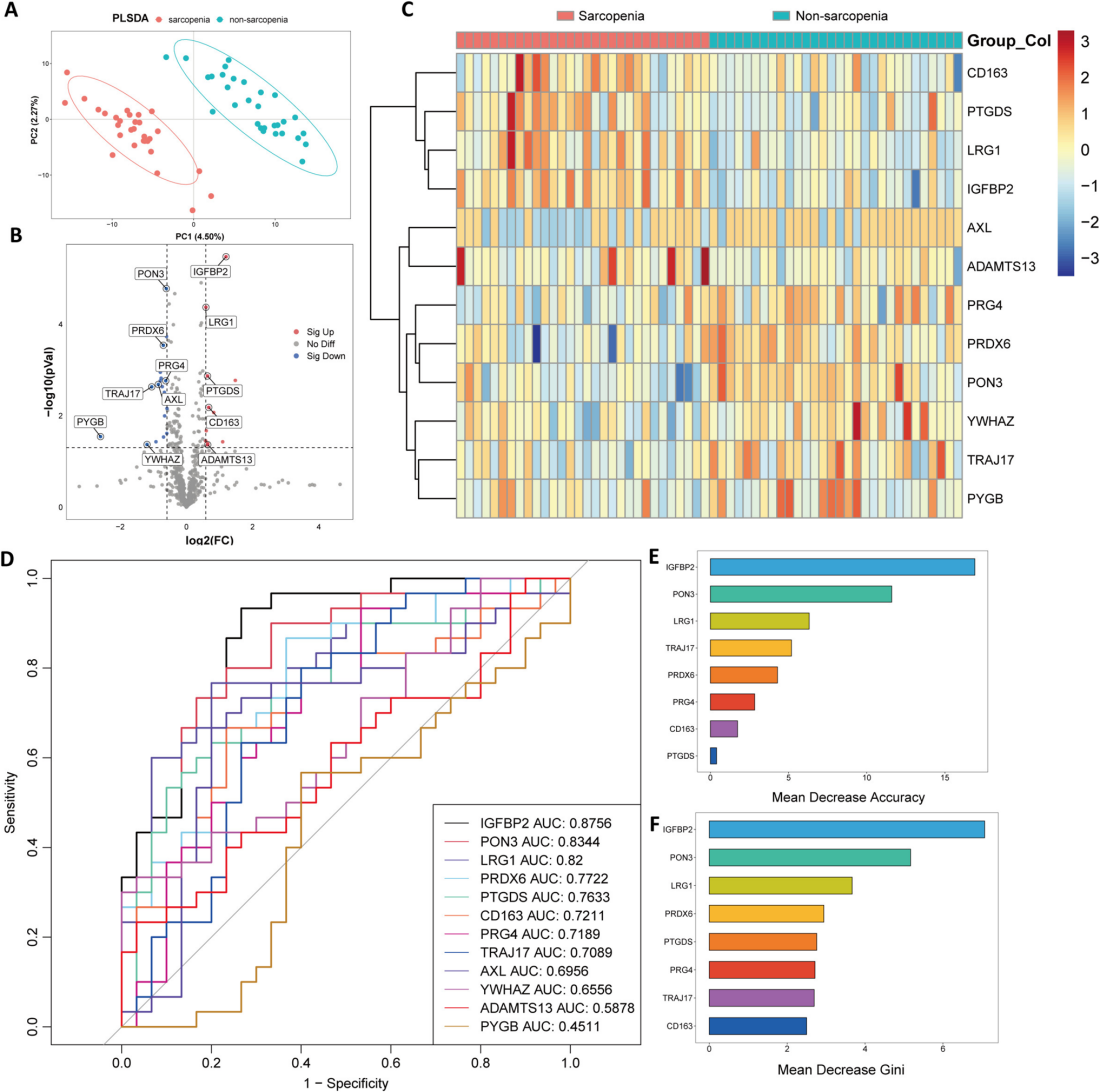
DIA analysis and DEPs selection. **A** Partial Least-Squares analysis of proteomic data. **B** Volcano plot of DEPs. Blue dots represented significantly down-regulated (FC < 0.667 and *p* < 0.05) proteins in the sarcopenic group; Red dots represented significantly up-regulated (FC > 1.5 and *p* < 0.05) proteins in the sarcopenic group; Grey dots represented non-significant proteins. **C** Hierarchical clustering heatmap of 12 DEPs. Proteins were shown in rows. Samples were shown in columns. Colour represented the expression levels between two groups. **D** ROC analysis of 12 DEPs to differentiate individuals with sarcopenia from those without sarcopenia, with AUC values listed in lower right. Eight selected DEPs were ranked in descending order of importance to the prediction accuracy based on mean decrease accuracy (**E**) and Gini index (**F**). *ROC* Receiver Operating Characteristic, *AUC* area under the ROC curve

DDA mode identified a total of 18,816 peptides and 2832 proteins. DIA-based analysis recognized 9207 peptides and 1127 proteins, with a false discovery rate (FDR) of less than 1%. Ultimately, 1,018 proteins were annotated and quantified (Supplementary Table 1). Partial Least-Squares regression analysis was performed on both sarcopenic and non-sarcopenic groups to examine within-group reproducibility and between-group differences (Fig. 1A for PLS-DA score plot, Supplementary Fig. 2 for validation model). The results indicated consistency within each group and a clear distinction between them.

### Identification of differentially expressed proteins

Compared with the non-sarcopenic group, a total of 39 DEPs were identified in the sarcopenic group, with a cutoff value of fold change > 1.5 (or < 0.667) and *p* < 0.05, including 17 up-regulated proteins and 22 down-regulated proteins (Fig. 1B, Supplementary Table 2). Following the exclusion of proteins: a) without a defined gene (n = 7), b) with an uncharacterized protein description (n = 1), and c) related to hemoglobin (n = 9) or immunoglobulin (n = 10), 12 proteins were selected for further analysis (Supplementary Table 3). Hierarchical clustering analysis of these 12 DEPs revealed different protein expression patterns between the sarcopenic and non-sarcopenic groups, with proteins of each group generally clustering together (Fig. 1C).

### ROC analysis and random forest test of DEPs

Receiver Operating Characteristic (ROC) analysis was performed to evaluate the potential diagnostic value of the 12 selected DEPs (Fig. 1D). Applying a threshold of AUC > 0.7, a total of 8 proteins, namely insulin-like growth factor-binding protein-2 (IGFBP2), paraoxonase-3 (PON3), leucine rich alpha-2-glycoprotein 1 (LRG1), peroxiredoxin 6 (PRDX6), prostaglandin D2 synthase (PTGDS), scavenger receptor cysteine-rich type 1 protein M130 (CD163), proteoglycan 4 (PRG4), and T cell receptor alpha joining 17 (TRAJ17), were selected for further investigation (Supplementary Table 4). IGFBP2 (AUC 0.8756) and PON3 (AUC 0.8344) demonstrated the top 2 AUC values, indicating their decent ability to differentiate individuals with sarcopenia from those without sarcopenia.

ROC model including these 8 proteins achieved an AUC value of 0.9533, with specificity of 0.933 and sensitivity of 0.833 (Supplementary Fig. 3A). ROC model enrolling IGFBP2 and PON3, the top 2 proteins, displayed an AUC value of 0.9022 (specificity 0.867, sensitivity 0.867) (Supplementary Fig. 3B). Random forest test was performed to evaluate variable importance of the selected proteins in sarcopenia prediction. Of them, IGFBP2 and PON3 were determined to be the top 2 crucial proteins, showing remarkably higher mean decrease accuracy (MDA) and mean decrease Gini (MDG) index (Fig. 1E and F, Supplementary Table 5).

### Associations between DEPs and clinical parameters

**Fig. 2:**
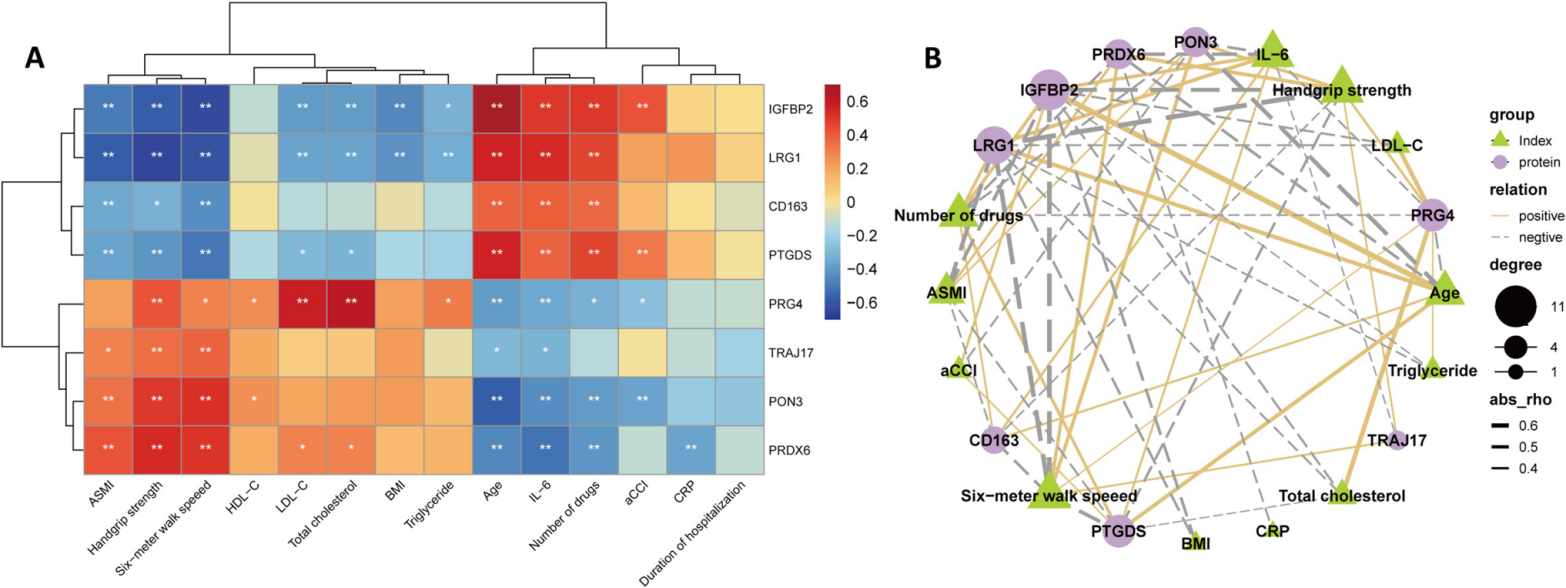
Correlation analysis. **A** Heatmap of correlations between DEPs and clinical parameters, *p < 0.05, **p < 0.01, ***p < 0.001. **B** Correlation network of significant correlations (*p* < 0.05). Degree represented the number of the nodes connected to the exact node, demonstrated by the size of the node. Purple circles represented DEPs. Green triangles represented the clinical parameters. The thickness of the lines represented the rho value. Yellow solid line represented a positive association. Grey dashed line represented a negative association

To investigate the associations between selected DEPs and clinical indices, Spearman’s correlation analysis was conducted, with results displayed in a heatmap (Fig. 2A, Supplementary Table 6). The significant associations were presented in a co-occurrence network (Fig. 2B). ASMI, grip strength, and six-meter walk speed were positively correlated with PRDX6, PON3, and TRAJ17, and inversely correlated with PTGDS, CD163, LRG1, and IGFBP2. aCCI was positively correlated with IGFBP2 and PTGDS, and negatively correlated with PON3 and PRG4. The number of drugs showed positive correlations with IGFBP2, LRG1, CD163, and PTGDS, with negative correlations with PRG4, PON3, and PRDX6. IL-6 demonstrated positive correlations with IGFBP2, LRG1, CD163, and PTGDS, and negative correlations with TRAJ17, PRG4, PON3, and PRDX6. HDL-C level exhibited positive correlations with PON3 and PRG4. Triglyceride level was positively correlated with PRG4, and negatively correlated with IGFBP2 and LRG1. LDL-C and total cholesterol both displayed positive correlations with PRDX6 and PRG4, and negative correlations with IGFBP2, LRG1, and PTGDS.

### DEPs functional enrichment and PPI network analysis

For further understanding of the mechanisms of plasma proteins in sarcopenia development, gene ontology (GO) enrichment, Kyoto encyclopedia of genes and genomes (KEGG) enrichment and Interpro (IPR) domains analysis were performed on these 8 proteins. GO analysis revealed significant enrichment in scavenger receptor activity, antioxidant activity, peroxiredoxin activity, arylesterase activity, and polysaccharide binding (Supplementary Fig. 4A and 4B, Supplementary Table 7). KEGG pathway analysis illustrated that the DEPs were mainly annotated with terms about Metabolic pathways and Arachidonic acid metabolism (Supplementary Fig. 4C and 4D, Supplementary Table 8). IPR enrichment analysis identified the enriched functional domains of DEPs, including Alkyl hydroperoxide reductase subunit C/Thiol specific antioxidant, Peroxiredoxin C-terminal, and Arylesterase (Supplementary Fig. 4E and 4F, Supplementary Table 9). Furthermore, a protein–protein interaction (PPI) network analysis was conducted to investigate the interactions between these proteins in sarcopenia (Supplementary Fig. 4G).

### Experimental validation of potential protein biomarker for sarcopenia

**Fig. 3:**
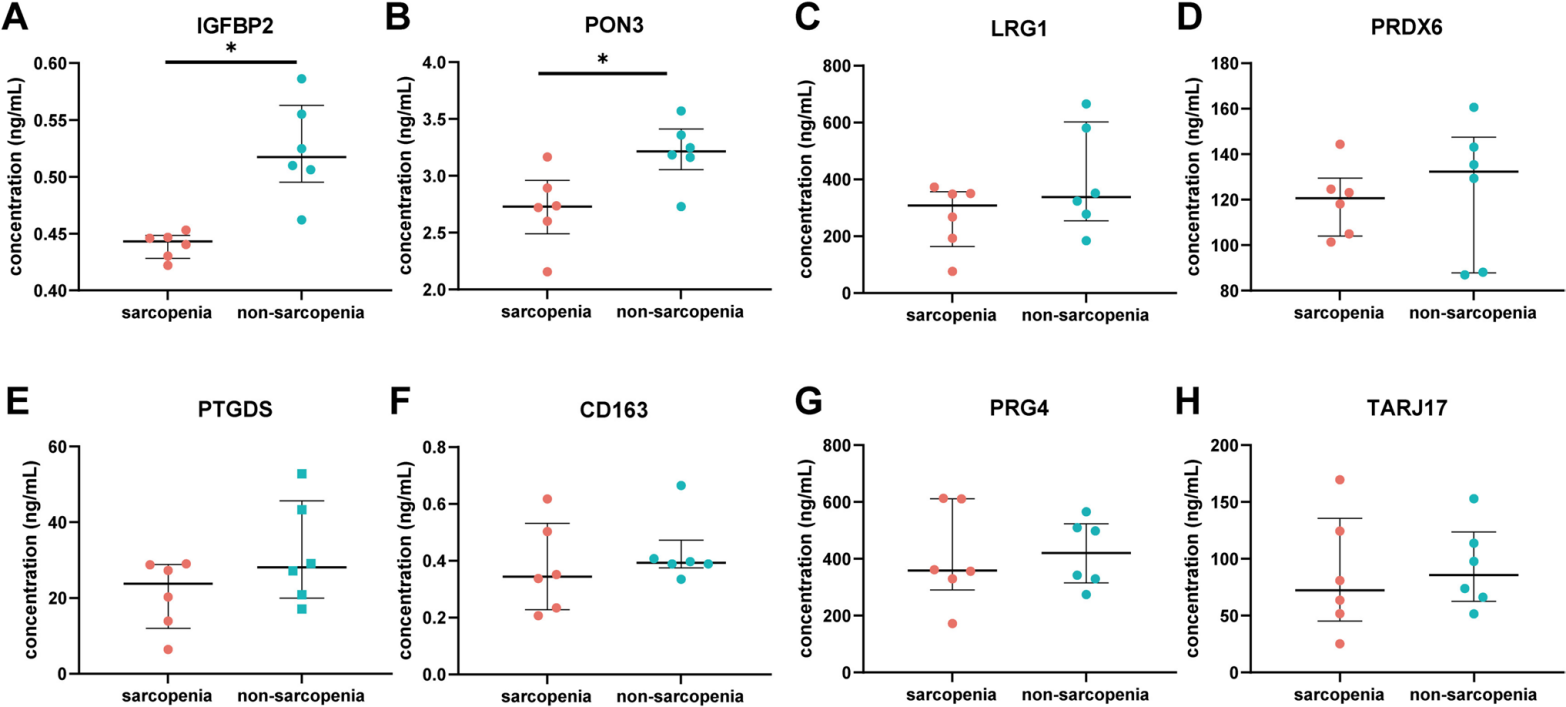
ELISA validation of selected DEPs in plasma samples of participants with sarcopenia (n = 6) and those without sarcopenia (n = 6). Protein concentrations determined by ELISA for **A** IGFBP2, **B** PON3, **C** LRG1, **D** PRDX6, **E** PTGDS, **F** CD163, **G** PRG4, and **H** TRAJ17. Results were expressed as “median (interquartile range)”. Rank sum test was performed (*p < 0.05)

ELISA validation was conducted for the 8 selected proteins. Plasma samples from 6 participants with sarcopenia and 6 without sarcopenia were utilized to quantify the concentration of these proteins, as detailed in Table 2 and depicted in Fig. 3. Notably, PON3 was significantly down-regulated in the sarcopenic group, consistent with the DIA analysis, indicating its potential as a biomarker for sarcopenia development. Although IGFBP2 showed significant differences between the two groups in ELISA validation, its regulation pattern was opposite to that observed in the DIA analysis. LRG1, PRDX6, PTGDS, CD163, PRG4, and TRAJ17 showed no significant differences between the sarcopenic and non-sarcopenic group in ELISA validation.

**Table 2:** Plasma concentration validation of selected proteins in participants with and without sarcopenia by ELISA

Protein (ng/mL)	Sarcopenia (n = 6)	Non-sarcopenia (n = 6)	Z-value	*p*-value
IGFBP2	0.4432 (0.4282, 0.4484)	0.5174 (0.4952, 0.5629)	2.882	0.004*
PON3	2.7285 (2.4897, 2.9605)	3.21561 (3.0542, 3.4118)	2.242	0.025*
LRG1	308.1870 (163.9352, 356.2098)	337.5315 (254.4637, 602.1790)	0.961	0.337
PRDX6	120.6250 (104.0819, 129.5493)	132.3493 (87.8002, 147.4762)	0.48	0.631
PTGDS	23.7686 (12.0484, 28.8587)	28.1447 (19.9521, 45.6905)	1.281	0.200
CD163	0.3446 (0.2277, 0.5318)	0.3928 (0.3752, 0.4721)	0.962	0.336
PRG4	358.7390 (290.6268, 611.8025)	420.3340 (315.7745, 523.6555)	0.32	0.749
TRAJ17	72.2865 (45.1982, 135.6611)	85.8205 (62.5182, 123.5002)	0.32	0.749

Rank sum test was performed (*p < 0.05)

## Discussion

Our study investigated the plasma proteome of individuals with age-related sarcopenia and identified 8 DEPs that showed promising predictive performance, including IGFBP2, PON3, LRG1, PRDX6, PTGDS, CD163, PRG4, and TRAJ17. Functional analysis revealed significant enrichment of scavenger receptor activity, arylesterase activity, and antioxidant activity, along with notable alterations in metabolic pathways. Subsequent ELISA validation in an independent cohort confirmed significantly different expression levels of PON3 and IGFBP2 between individuals with and without sarcopenia. PON3 was consistently down-regulated in the sarcopenic group across both proteomic analysis and validation, while IGFBP2 showed inconsistency, being up-regulated in the sarcopenic group in proteomics but down-regulated in validation. Consistent with prior research, we found distinct plasma proteomic profiles between participants with and without sarcopenia. We identified sarcopenia-associated proteins that were not reported in the literature and observed proteins with differences from existing research findings, which may be related to various factors such as the geographic and ethnic backgrounds of the participants. Ultimately, we propose PON3 as a potential biomarker for age-related sarcopenia.

Paraoxonase-3 (PON3), the most recently discovered and the least studied protein of the paraoxonase family (PON1-3) [36], is mainly located in the mitochondria, endoplasmic reticulum and circulating HDL particles [37]. Current evidence suggests that PON3 participates in the pathogenesis of various diseases, primarily through its significant antioxidant properties [36, 38, 39]. HDL-associated PON3 also helps prevent LDL oxidation and inactivate oxidized LDL [40]. In the field of aging, PON family is involved in senescence and age-related diseases, with a focus on their antioxidative potential [41]. Oxidative stress is one of the key mechanisms in aging and sarcopenia [42–44]. Researchers observed a decline in PON1 level with increasing age [45]. Additionally, the rs662 polymorphism in the PON1 gene demonstrated a significant association with longevity, potentially attributed to its ability to enhance the antioxidant capacity of PON1 [46]. However, research on the role of PON3 in muscle aging is limited. Our study is the first to report the role of PON3 as a biomarker for sarcopenia in aged population. The correlation analysis revealed that PON3 was positively correlated with HDL-C level and muscle parameters. It could suggest that a decline in PON3 may lead to an overload of oxidative stress in skeletal muscles, which eventually contributes to sarcopenia onset and progression. Restoring PON3 level in aged individuals may reinstate the antioxidant activity and alleviate oxidative stress in skeletal muscles, eventually improving muscle mass and function.

Insulin-like growth factor-binding protein-2 (IGFBP2) is a member of the insulin-like growth factor-binding protein family (IGFBP1-6) [47]. Recent studies have established links between IGFBP2 and muscle cachexia [48], low skeletal muscle mass [17, 49] and decline in muscle strength and function [50]. In the proteomic analysis, IGFBP2 was found to be significantly up-regulated in individuals with sarcopenia. However, subsequent ELISA validation showed a low concentration of IGFBP2 in the plasma from the sarcopenic group. Given the demographics of our study cohort and the characteristics of IGFBP2, we propose that this finding may be attributed to two potential factors.

First, discrepancies were observed between the two cohorts. According to previous research, IGFBP2 was significantly associated with age [51], senescence [52], inflammation status [53–55], and various diseases [56–58]. In the cohort for proteomic analysis, compared with those without sarcopenia, individuals with sarcopenia exhibited older age, higher inflammatory levels, higher aCCI, and higher number of drugs, which could influence IGFBP2 expression level. Conversely, the cohort for ELISA validation exhibited balanced baseline characteristics. These discrepancies could account for the inconsistent results of IGFBP2. It is unrigorous to conclude a definitive association between IGFBP2 and sarcopenia based on a limited sample size and a single set of results. Studies with larger sample sizes and different validation methods are necessary.

Second, the possible existence of protein modifications of IGFBP2 and the antigen–antibody binding characteristics of sandwich ELISA should be considered. A targeted analysis approach may fail to replicate proteins identified by an untargeted method due to different detection mechanisms [59]. While DIA-based proteomics is an untargeted technique that identifies proteins by analyzing fragmented peptides, ELISA is a targeted technique that quantifies the specific epitope recognized by the antibody [59]. It has been noted that ELISA is not sensitive enough to quantify modified proteins, including post-translational modifications (PTMs) [60], isoforms, and complexes with other circulating plasma proteins [61, 62]. Since modified epitopes may affect the success of recognition or the effectiveness of binding of antibodies to antigen, ELISA quantification result depends on the antibody affinity of specific protein forms rather than its total amount. Researchers found age-specific protein modifications in dysfunctional aged skeletal muscles [63, 64]. Furthermore, IGFBP2 has been observed in phosphorylated [65], complexed [66–68], or fragmented forms [68] during pathophysiological processes. Thus, we speculate that IGFBP2 might undergo various modifications during sarcopenia pathogenesis, which may alter the targeted antigenic epitope for recognition and affect the affinity of antigen binding, ultimately resulting in detection discrepancies.

This is the first study revealing plasma biomarkers in Chinese hospitalized older adults with sarcopenia, with two independent cohorts for exploration and validation. PON3 has been identified as a potential novel biomarker for age-related sarcopenia. However, there are several limitations to consider. First, this cross-sectional study lacks longitudinal data on biomarker levels, emphasizing the need for longitudinal studies to elucidate dynamic changes of biomarkers throughout the pathogenetic process of sarcopenia. Second, the imbalanced baseline characteristics of the participants in the DIA analysis suggested the presence of potential confounding factors that could influence the outcomes. Despite experimental validation, the results, particularly regarding IGFBP2, should be interpreted cautiously and warrant further validation. Third, recruiting hospitalized individuals with sarcopenia limits the generalizability of our findings to community-dwelling and nursing home residents. Multi-center and multi-regional research are necessary to identify specific biomarkers for individuals with sarcopenia with various characteristics. Fourth, this study provided preliminary validation of sarcopenia biomarkers but lacked insight into the underlying mechanisms. Considering the perspectives of criteria for aging biomarkers [69], functional validation and mechanism elucidation through cellular experiments, animal models, and clinical trials should be carried out. Additionally, beyond protein abundance, the specific protein modification forms, such as phosphorylation, ubiquitination, and methylation, should be detected and validated using updated technologies.

## Conclusion

In conclusion, this cross-sectional study applied proteomic analysis and experimental validation to explore the sarcopenic proteome and to identify candidate biomarkers for age-related sarcopenia. Our findings indicated a distinguishable proteome between individuals with and without sarcopenia. PON3 was confirmed to be down-regulated in older adults with sarcopenia, which may lead to sarcopenia due to impaired antioxidant defenses. For IGFBP2, the discrepant result suggested a possible modification of IGFBP2, and further studies are needed to confirm this hypothesis. We propose that PON3 could be considered as a potential biomarker for age-related sarcopenia and a promising target for intervention. Further cellular, animal and clinical studies of PON3 are needed for an in-depth understanding of the pathogenesis and mechanism of sarcopenia.

## Supplementary Information

Below is the link to the electronic supplementary material.

Supplementary file1 (DOCX 236 KB)

Supplementary file2 (XLSX 739 KB)

## Data Availability

The proteomics data have been deposited to the ProteomeXchange Consortium (https://proteomecentral.proteomexchange.org) via the iProX (https://www.iprox.cn/) partner repository with the dataset identifier PXD053735.
