# Supplementary material for: Plasma biomarkers in patients with age-related sarcopenia: a proteomic exploration and experimental validation
Source: Aging Clin Exp Res. 2024 Dec 27;37(1):13. doi: 10.1007/s40520-024-02903-7 (PMC11671435; doi:10.1007/s40520-024-02903-7)
Supplement: Supplementary file 1 — Supplementary file1 (DOCX 236 KB) [file 40520_2024_2903_MOESM1_ESM.docx]

**Supplementary Figures**

**Journal: Aging Clinical and Experimental Research**

**Plasma Biomarkers in Patients with Age-related Sarcopenia: A Proteomic Exploration and Experimental Validation**

Qinqing Lin^12^, Kangyong Li^34^, Liwei Li^1^, Lichang Guan^1^, Yingtong Zeng^3^, Dake Cai^3^, Jing Zhou^13^*, Lishu Xu^15^*

1 Department of Geriatric Gastroenterology, Guangdong Provincial People's Hospital (Guangdong Academy of Medical Sciences), Southern Medical University, Guangzhou, China.

2 Shantou University Medical College, Shantou, Guangdong, China.

3 Department of Pharmacy, Guangdong Provincial People's Hospital (Guangdong Academy of Medical Sciences), Southern Medical University, Guangzhou, China.

4 Guangzhou University of Chinese Medicine, Guangzhou, China.

5 Guangdong Provincial Institute of Geriatrics, Guangdong Provincial People's Hospital (Guangdong Academy of Medical Sciences), Southern Medical University, Guangzhou, China.

*Correspondence to

Lishu Xu, xulishu@gdph.org.cn

Jing Zhou, zhoujing@gdph.org.cn


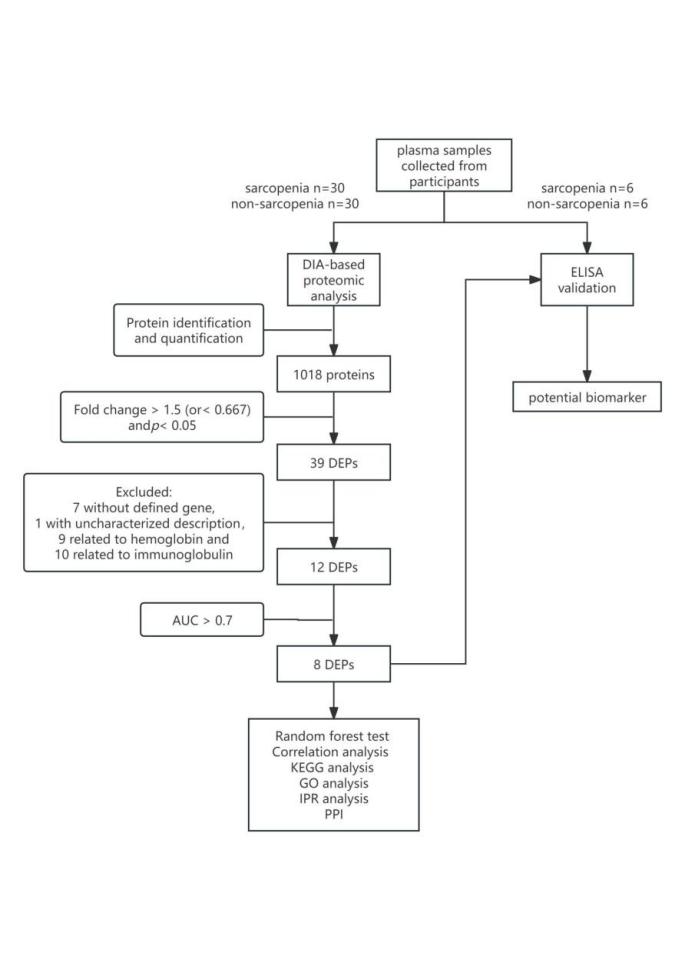


Supplementary Figure 1. Flowchart of the study design for sarcopenia biomarker screening and validation. GO: Gene Ontology; KEGG: Kyoto Encyclopedia of Genes and Genomes; KOG: Eukaryotic Orthologous Groups; PPI: protein-protein interaction; AUC: area under the curve.


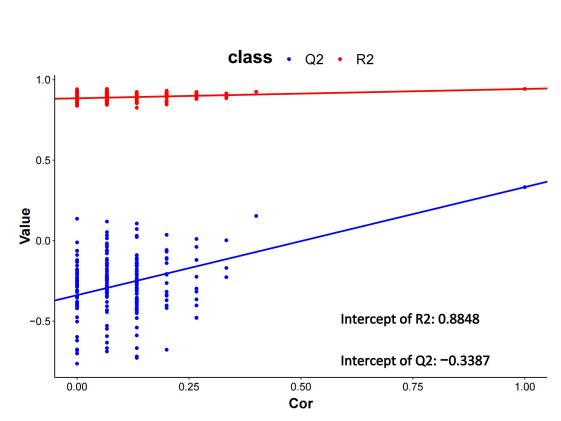


Supplementary Figure 2. PLS-DA validation model through 200 permutation tests.


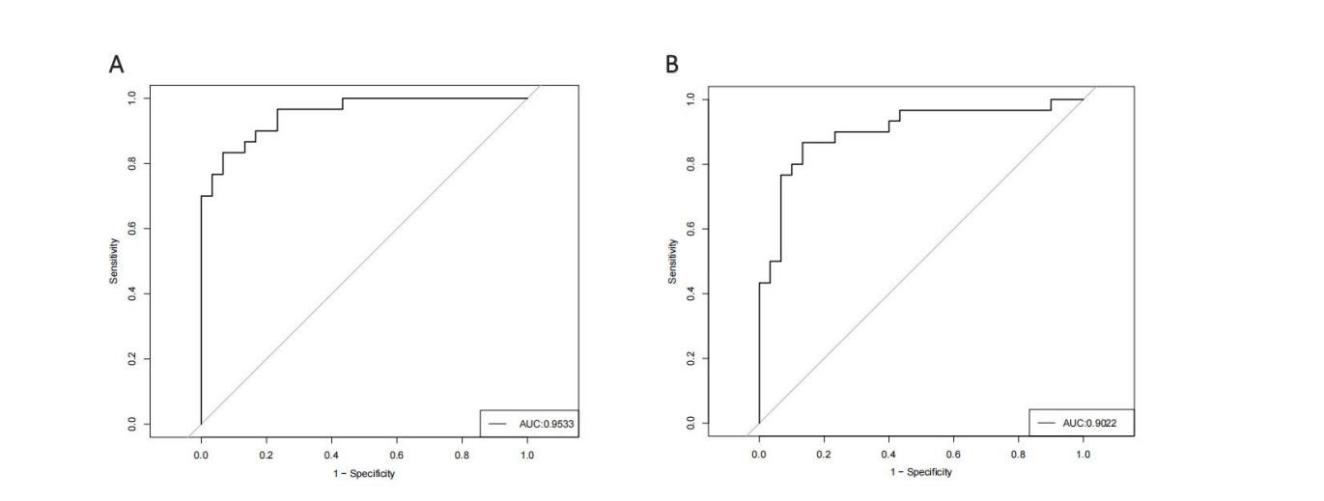


Supplementary Figure 3. (A) ROC analysis of 8 selected proteins; (B) ROC analysis of IGFBP2 in combination with PON3.


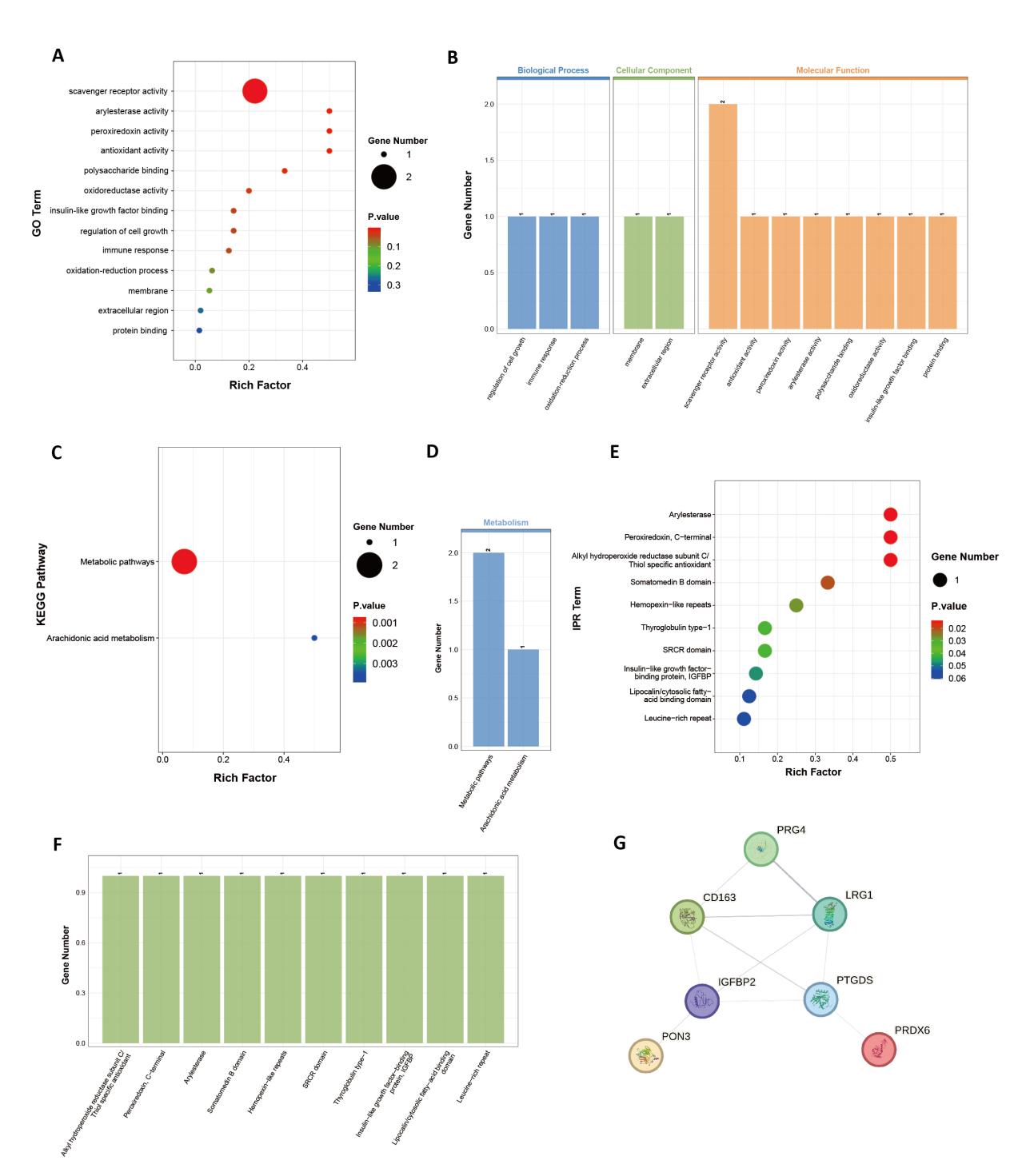


Supplementary Figure 4. Functional analysis and Protein-protein interaction network analysis. GO functional enrichment analysis presented in scatter plot (A) and bar chart (B). KEGG pathway analysis presented in scatter plot (C) and bar chart (D). IPR analysis presented in scatter plot (E) and bar chart (F). The size of the circle represented the number of DEPs in the GO/KEGG/IPR term. The color of the circle represented the *p*-value for the enrichment degree. SRCR, scavenger receptor cysteine-rich. (G) Protein-protein interaction network analysis. Nodes were filled with the known or predicted three-dimensional structures of the proteins. Edges represented protein-protein associations. Confidence (i.e. the strength of data support) was represented by the thickness of association line. Minimum required interaction score was set as confidence of 0.150.
